# The deep population history in Africa

**DOI:** 10.1093/hmg/ddab005

**Published:** 2021-01-12

**Authors:** Nina Hollfelder, Gwenna Breton, Per Sjödin, Mattias Jakobsson

**Affiliations:** Human Evolution, Department of Organismal Biology, Uppsala University, Norbyvägen 18C, 75236 Uppsala, Sweden; Human Evolution, Department of Organismal Biology, Uppsala University, Norbyvägen 18C, 75236 Uppsala, Sweden; Human Evolution, Department of Organismal Biology, Uppsala University, Norbyvägen 18C, 75236 Uppsala, Sweden; Human Evolution, Department of Organismal Biology, Uppsala University, Norbyvägen 18C, 75236 Uppsala, Sweden; Palaeo-Research Institute, University of Johannesburg, Physical, Cnr Kingsway & University Roads, Auckland Park, Johannesburg 2092, South Africa; SciLifeLab, Stockholm and Uppsala, Entrance C11, BMC, Husargatan 3, 752 37 Uppsala, Sweden

## Abstract

Africa is the continent with the greatest genetic diversity among humans and the level of diversity is further enhanced by incorporating non-majority groups, which are often understudied. Many of today’s minority populations historically practiced foraging lifestyles, which were the only subsistence strategies prior to the rise of agriculture and pastoralism, but only a few groups practicing these strategies remain today. Genomic investigations of Holocene human remains excavated across the African continent show that the genetic landscape was vastly different compared to today’s genetic landscape and that many groups that today are population isolate inhabited larger regions in the past. It is becoming clear that there are periods of isolation among groups and geographic areas, but also genetic contact over large distances throughout human history in Africa. Genomic information from minority populations and from prehistoric remains provide an invaluable source of information on the human past, in particular deep human population history, as Holocene large-scale population movements obscure past patterns of population structure. Here we revisit questions on the nature and time of the radiation of early humans in Africa, the extent of gene-flow among human populations as well as introgression from archaic and extinct lineages on the continent.

## Introduction

Africa has been identified as the place of origin of *Homo sapiens*, evident by the highest genetic diversity and deepest splits being found among people from this continent. The fact that the earliest fossil finds of early humans have been found in Africa, including northern (dating to about 300 000 years old), southern (a 260 000-year-old individual) and eastern Africa (about 195 000–160 000 years old, 1–7) further points to Africa’s importance for early human development. The African fossil record of early humans displays a large morphological diversity and possible coexistence with now-extinct lineages of other *Homo* species or forms (2,5–15). The complex early history of humans in Africa poses questions on how our species emerged, the deep population structure and its origin, and whether there has been a genetic exchange of deeply structured and/or archaic populations with modern humans in Africa. With more genomes becoming available from diverse African groups as well as efforts in ancient DNA sequencing, we are gradually gaining more information on African diversity and these genomic data can reveal unknown ancestries or genetic make-up by providing information before migration or admixture events and can give indications of population continuity.

Hunting and gathering were the lifestyle of all humans prior to the Neolithic transition and emergence of agriculture and pastoralism. Most hunter-gatherer groups have been replaced or incorporated in expanding agriculturalist and pastoralist populations (16), only a few extant groups continue to practice this way of life and can be found scattered in various regions across the globe, including Africa. Today, most remaining practicing hunter-gatherer populations occupy geographical areas that are not suitable for agriculture or grazing, such as rainforests and deserts. As a consequence, the number of hunter-gatherer groups (and practicing individuals) has been reduced drastically in the last few thousand years, due to the expansion and increase in population size of agriculturalists and pastoralists (16–21).

In Africa, hunter-gatherer groups can be found in the south and east and in the Congo Basin. In southern Africa, the San hunter-gatherers and the Khoekhoe herders, who adopted a pastoralist lifestyle after contact with east African pastoralists (22), inhabit arid regions unsuitable for agriculture. They are collectively named Khoe–San, in correspondence to speaking Khoisan languages, which comprise ‘click’-rich languages belonging to five linguistic families that are otherwise unrelated (23). In eastern Africa, there are various populations that practice, or have until recently, a hunter-gatherer lifestyle. East African hunter-gatherers (EAHG) have been shown to be more closely genetically related to each other than to other hunter-gatherer populations in Africa (24–27). Furthermore, several hunter-gatherer populations inhabit the rainforests of equatorial Africa, such as the Biaka, Baka, Bakola, Bedzan, Batwa, Twa and Mbuti. These rainforest hunter-gatherer (RHG) groups have adopted the languages of neighboring agricultural populations (27,28). The rapid expansion in the last 5000 years, as well as other recent migrations in Africa obscure patterns of deep population structure in sub-Saharan Africa (20) and much of sub-equatorial Africa, is today populated by people of western African descent speaking Bantu languages.

It is now increasingly recognized that the human population in Africa was stratified prior to the migrations of humans Out-of-Africa some 80 000 years ago (ya), which rejects the idea of a single panmictic human population in eastern Africa that expands into southern and western Africa and out from Africa (20,29,30). To investigate deep population structure, that goes beyond 100 000 years, it is essential to investigate populations from a wide geographic range that potentially represent descendants of groups living in the area prior to new groups arriving in the second half of the Holocene. Hunter-gatherer populations and DNA samples from ancient individuals can provide insight into deeper population history that is not obscured by the confounding factors of recent large-scale migrations. Recent admixture can, for example, lead to an underestimation of split times (31). Ancient DNA also offers the possibility of unearthing genetic diversity that is lost in the modern gene-pool. With the rapid development in genome sequencing technologies, the number of whole-genome sequences available from modern-day hunter-gatherers and genome-wide information from African archeological samples has increased rapidly in recent years, despite the fact that much of Africa has not been extensively surveyed by archaeologists and that DNA does not preserve particularly well in the hot and humid climate. Since the publication of the first ancient genome from east Africa in 2015 (32), genome-wide information from several ancient African samples was successfully retrieved, with samples spanning the entire continent and even African islands; the oldest samples, from north Africa, date to 15 000 years (31,33–40).

## Hunter-gatherer Population Structure

The high genetic diversity in Africa is a result of a deep population structure, likely shaped by periods of isolation, possibly driven by climatic oscillations (30,41). Various measurements of genetic diversity show the most extreme values in Africa, such as comparatively high allelic richness, heterozygosity as well as short runs of homozygosity (42). African genomes, on average, harbor the most divergent genetic lineages among all humans, with the exception of lineages tracing to Neanderthals and Denisovans (19–21,42). African hunter-gatherer groups have previously been shown to be the most genetically diverse contemporary populations, carry the most basal uniparental markers (24,43–46), and harbor the deepest autosomal branches (18,25,29,31,42,47–50).

The genetic relationship between the various contemporary regional hunter-gatherer groups can be modeled with isolation-by-distance, revealing that hunter-gatherer populations were connected, potentially in overlapping areas and inhabited larger geographic areas (35,40,51). There are indications of gene-flow between Khoe–San and other African hunter-gatherer populations after they split from each other (27,35,42,52), suggesting that the genetic exchange persisted until the Holocene. For instance, there appear to have been a clinal link between southern African San groups and eastern African hunter-gatherers, where prehistoric individuals from e.g. current-day Malawi display affinities to both San groups and to eastern African hunter-gatherers (35,38), with some San-like ancestry, detected in ancient individuals excavated in eastern Africa (40). Today, the eastern African hunter-gatherers display low *N_e_* due to a small census size (27,53).

The contraction of once wide-spread, overlapping hunter-gatherer populations, which led to the scattered populations we know today, left a mark in their genomes. For instance, many hunter-gatherer populations do not display an effective population size (*N_e_*) increase in the Holocene, contrasting to many other groups (35,40,42). Today the RHG exhibits lower *N_e_* than surrounding agriculturalists (54). The southern African Khoe–San have repeatedly been shown to display the highest genetic diversity in global comparisons (18,29,31,42,48). This is caused by a large *N_e_* of the Khoe–San for the majority of human history, as well as admixture from non-Khoe–San populations (18,19,42). The lineage leading to current-day Khoe–San populations likely inhabited a larger geographical region than the distribution of contemporary southern African hunter-gatherers (35,40). It has been hypothesized that the ancestors of the Khoe–San were most likely the only inhabitants of southern Africa for most of the region’s prehistory (31,35). *N_e_* starts to differ between Khoe–San and all other groups some 300 000–200 000 ya (42,52), demonstrating that there was population structure at that time, followed by a population decline that affected populations differently. For instance, the San and RHG maintained a larger *N_e_* than other African populations prior to 60 000 ya, but all groups, including the Khoe–San and RHG, display population decline in this time period (42,52).

Populations from north Africa are often exempted when discussing deep African history as they show mainly Eurasian ancestry, with a modest ancestry component assigned to sub-Saharan Africa (55,56). However, recent ancient DNA results from Moroccan fossil samples dating back 15 000 years provide insights into hunter-gatherers from north Africa. These samples derive most of their ancestry from a non-African like source (best match: Natufian) but also derive one-third of their ancestry from sub-Saharan Africa (37). The sub-Saharan ancestry component appears as a mix of east and west African ancestry but there are no clear sources for these ancestries, rather the ancestry is likely from an unsampled population that is related to both modern-day west and east Africans. These individuals from Morocco along with younger samples (7000 and 5000 years old) show decreasing sub-Saharan ancestry over time (36,37), a trend which has also been observed in Egypt (34). This pattern likely arises from isolation in the Maghreb since the Upper Paleolithic (36). Whether the non-African ancestry derives from an early back-admixture event or if there is long-standing gene-flow between north Africa and non-African populations has not yet been resolved.

## Models of Human Evolution and Population Divergence Time Estimates

It is common to model the evolutionary history of humans as a bifurcating tree and to estimate divergence times between the branches. Trees are a simplification and they miss some features of human population history, such as gene-flow, but they are useful as models in order to understand the relations among groups and the relative divergences among groups. The estimates for a specific event typically vary due to the methods and the assumptions of the models, the scaling parameters (e.g. mutation rate and generation time), and the set of individuals and populations used for the comparisons (57). Alternatively, human evolution can be represented with metapopulation models (58), though such modeling for addressing questions of deep human history is still rare (59).

**Figure 1:**
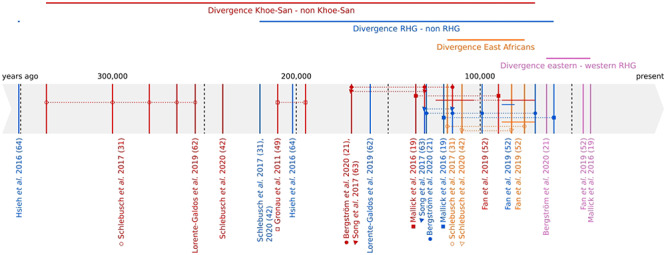
Schematic overview of divergence times inferred from whole-genomes. Estimates of the divergence between Khoe-San and other populations are represented in red; between rainforest hunter-gatherers and other populations in blue; between east African hunter-gatherers and other populations in orange; and between eastern and western rainforest hunter-gatherers in pink. Point estimates from the same study and for the same event are identified by a symbol and are connected by dotted lines; a given study can give several point estimates because e.g. of different comparative populations or different methods. In the case of Song *et al*. (63), estimates were given as ranges, which are represented by a continuous line.

Figure 1 gives an overview of the range of estimates of deep divergence events in human population history based on high-coverage autosomal genomes from a range of studies (see 57 for the underlying data). Note that estimates based on MSMC and MSMC2 methods (60,61) are more recent than estimates based on e.g*.* genealogical concordance, site-frequency based methods or approximate Bayesian computation analyses (after rescaling with the same mutation rate), potentially due to the fact that the former methods use the midpoint of cross-coalescences as the divergence time estimate. However, the rank order of divergence times does not differ much between the different approaches, which points to consistent population topology across the methods. The divergence between the ancestors of the Khoe–San and the ancestors of the rest of modern humans is estimated to between 340 000 and 200 000 ya (31,42,49,62), and with younger estimates (160 000–90 000) based on the MSMC cross-coalescence approach (19,21,52,63). The next event assuming a simplified bifurcating tree is a divergence between the RHG ancestors and the ancestors of the rest of modern humans (minus the Khoe–San); the estimates vary from 350 000 to 70 000 ya (19,21,31,42,52,62–64) but are generally more recent than the Khoe–San divergence. Eastern African groups, including hunter-gatherers, such as the Hadza and the Sandawe, point to divergences from all other African groups, including western Africans, at ~140 000–70 000 ya (31,42,52).

**Figure 2:**
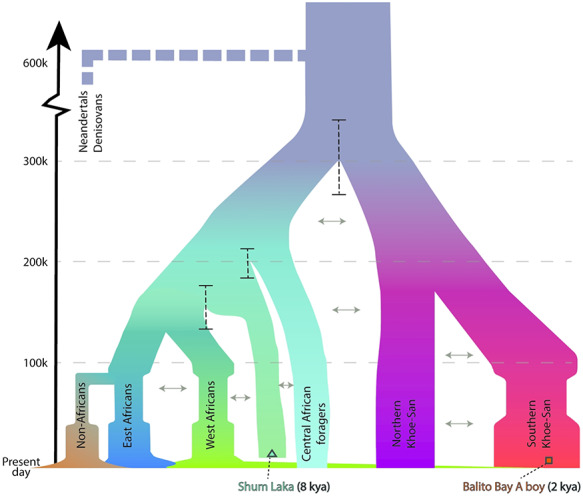
Demographic model of African history and estimated divergences. Summary of population split times, hierarchy and population sizes (29,31,39,42,54). Vertical dashed black lines indicate confidence intervals of population split times estimated using the ‘TT method’ (84). To estimate the divergence times between the Shum Laka individual, we download the genome sequence data (39) and used the ‘TT’ method to compute divergence times. The ‘TT’ method uses sample configurations of variants across the genome in two individuals in a population divergence model. Independent of the drift in each daughter population and given a mutation rate, this approach estimates the number of generations since a population divergence for a pair of individuals.

Most studies find a bifurcation as the deepest divergence among humans (but see next section for the potential impact of unknown groups). For instance, mtDNA and complete genomes repeatedly find the deepest divergence among humans being between Khoe–San and all other groups (20,21,31,42,49). However, some studies point at alternatives, for instance, a very deep divergence involving RHGs (64) or a trifurcation as the deepest divergence, partly based on genetic data from 8000 years old human remains from west Africa (Shum Laka), between Khoe–San, west Africans and RHGs (39). The 8000 years old Shum Laka individual interestingly showed both west African and RHG ancestry, and if we estimate the divergence time for this individual, it shows divergence time to the Khoe–San of 260 000–350 000 years, and 120 000–220 000 years to west Africans and RHGs (Fig. 2), solidly placing the 8000 years old Shum Laka individual as an individual on the non-Khoe–San branch, related to west Africans and RHGs (as a result of admixture or shared ancestry).

Although not as well studied, the estimates for the divergence between northern and southern San have a large range as well, from ~170 000 to ~30 000 ya (19,31,52). This possibly reflects the fact that an isolation-by-distance model might be a better representation of the relationships between these populations (51). The common origin of western and eastern RHG has been demonstrated (65) and the divergence between western and eastern RHG has been estimated to ~60 000–40 000 ya (19,21,52). Note that all these estimates are based on MSMC/MSMC2, which typically provide more shallow divergence time estimates. See Figures 1 and 2 for an overview of the human population topology and divergence time estimates.

## Introgression from Extinct Lineages

There has been a resurging interest in the question of archaic admixture in Africa. With the recovery of the first genome-wide data from Neanderthals and Denisovans (66,67), admixture from these archaic groups has become evident in non-Africans. With new methods that do not necessarily require a reference genome from the introgressing lineage (62,68–71) and better genome data, it is now possible to investigate the question of introgression from extinct lineages in Africa. Given the rich human and hominin history in Africa, the lineage leading to modern-day humans has the potential to interact and also mix with hominin groups that separated from our lineage far back in time, perhaps on par with the divergence of Neanderthals and Denisovans some 600 000 ya. It is also likely that distinct modern human groups existed, possibly diverging a few hundred thousand years ago or more recently, that later disappeared for some reason. Such populations could have mixed with human groups that did contribute substantially to today’s people in Africa. We can refer to this latter phenomenon as introgression from ‘ghost’ populations, and the former case as ‘archaic’ introgression. One way to separate these two introgression processes is to define ‘archaic’ introgression as involving a hominin group separated from the human lineage before the deepest divergence among modern humans, some 300 000 ya, and ‘ghost’ introgression involving extinct anatomically modern groups that separated around 300 000 ya or more recently.

Since the publication of the Neanderthal genome (67), the Neanderthal admixture into human populations is continuously being unraveled, which also has bearing on the understanding of the demographic history of African populations. The west African Yoruba have often been used as an unadmixed reference population to estimate global Neanderthal admixture proportions (67), although they have been shown to carry small levels of Neanderthal ancestry (16,72). A new method that eliminates the need for an unadmixed modern source showed that the Neanderthal ancestry proportion in Africans was larger than previously thought (IBDmix, 72). Chen *et al*. (72) found on average 17 Mb of Neanderthal sequence per individual of which 94% is shared with non-Africans. Bergström *et al*. (21) also find archaic variants from Neanderthal and Denisovans in west Africans that are absent in non-Africans, reflecting the larger genetic variation found in Africa. The Neanderthal ancestry in modern Africans can be explained by back migration from an ancestral European-like source into Africa (72), a scenario which is supported by other studies that found Neanderthal ancestry proportions to correlate with Eurasian ancestry proportions (21,32,73,74).

Even before the first Neanderthal genome was published, signals of introgression from deeply branching populations had already been detected in African populations (68,75). A potential explanation for this observation would be that these other populations are now extinct and their genetic material only lives on as traces in contemporary populations. With the publication of the Neanderthal and Denisovan genomes (66,67), it became evident that while Neanderthals and Denisovans can account for all of the archaic admixture in non-Africans, they cannot account for the signals observed in African populations. With the absence of representative reference genomes, archaic and ghost introgression is often identified as divergent regions in the genome that contain strongly linked ancestral variants and/or show deep coalescence times. The sequences identified in this way might stem from an unsampled extinct ‘ghost’ population, or represent patterns arising from some alternative complex demography, that include deep stratification among past groups. However, with the analyses presented this far, it is difficult to differentiate between deep structure, real introgression from archaic and/or ghost populations, and statistical artifacts (76,77).

Admixture from ‘ghost’ populations and even ‘archaic groups’ has been suggested to explain particular patterns of variation in various African populations (31,35,39,62,68–71,75,78–82) (Table 1). It has been hypothesized that interbreeding with archaic populations happened frequently but at low levels (80). Especially west Africans have often been identified to harbor archaic ancestry. This signal has been shown to come from one or more populations that split from the ancestors of modern human approximately at the same time or slightly earlier than the Neanderthal split (35,39,62,68–71,75,81). Another signal, or an alternative explanation to the archaic introgression, has been proposed for west Africans, with introgression from a ghost population which split around 300 000 ya (31,39). The admixture signal from an archaic or ghost population in west Africa is consistent with the non-symmetrical relationship of the Khoe–San populations to west and east African populations (35). Furthermore, the differences in archaic ancestry proportions across Africa indicate that population structure can already have been established at the time of introgression, or alternatively, this observation can be a consequence of gene-flow from non-Africans (72). Some studies have also suggested that the introgressing sequences come from a population with a large *N_e_*, which hints at it being a structured population itself (69,71). Another possibility is that the large *N_e_* is shaped by multiple introgression events from divergent lineages, which are hard to distinguish without archaic reference sequences. Unfortunately, many studies of archaic or ghost introgression in Africa focus on a few populations and/or use only one method for inference, so that the effect of the identified archaic or ghost introgression is not yet comparable across all major branches of modern humans in a systematic way.

**Table 1:** Overview archaic and ‘ghost’ introgression

Publication	Split time of source population	Admixture recipient	Modern admixed populations	Introgression time	Admixture proportion (%)	Method
Plagnol and Wall (2006), (68)			Yoruba		5	S^*^
Wall *et al*. (2009), (75)			Yoruba			S^*^
Wall *et al*. (2019), (82)		ancestors of KhoeSan	African populations included in the GenomeAsia 100K project			LD-based
Speidel *et al*. (2019), (70)			Yoruba			Relate
Hsieh *et al*. (2016), (80)	1.08 mya		Biaka, Baka	9048 ya		S^*^
Durvasula and Sankararaman (2020), (71)	625 kya		Yoruba, Esan, Gambian, Mende	43 kya	11	CSFS
Hammer *et al*. (2011), (78)	700 kya		Biaka Pygmy, Mbuti Pygmy and San	35 kya	2	S^*^
Skoglund *et al*. (2017), (35)	Pre South African/other Africans split		Mende		13	qpgraph
			Yoruba		9	
Ragsdale and Gravel (2019), (81)	460−540 kya	Pre-split OOA branch	YRI	90−160 kya	4.7–9.2	Multi-population two-locus diversity statistics
			CEU, CHB		1.9–6.6	
Lipson *et al*. (2020), (39)	~KhoeSan/RHG split	Ancestral west African branch	Lemande, Yoruba, Mende, Bantu		10	qpgraph
			Mende		Additional 3	
		Mota (ancient east African)			29	
	~Neanderthal/*H. sapiens* split	Ancestral west African lineage	Lemande, Yoruba, Mende, Bantu		2	
			Mende		Additional 1	
Lorente-Galdos *et al*. (2019), (62)	528 kya		KhoeSan		3.8	ABC-DL
			Mbuti		3.9	
			Mandenka		5.8	
Lachance *et al*. (2012), (79)	1.2-1.3 mya		Hadza, Sandawe, WRHG	Predating the split of the populations		S^*^
Schlebusch *et al*. (2017), (31)	Pre ancient south African/ancient east African split		Yoruba		31	qpgraph
Hey *et al*. (2018), (69)	850 kya	Ancestral branch of Yoruba, Baka, Hadza and Sandawe	Yoruba, Baka, Hadza, Sandawe			IMa3

The table gives an overview of all studies that have found archaic admixture or admixture from a ‘ghost’ source in African populations to date. ‘Admixture recipient’ shows which branch experienced the introgression event while ‘modern admixed populations’ show the populations that were investigated and showed the admixture signal.

Interestingly, many studies identified a fairly recent time for the introgression from extinct lineages in Africa, with introgression events even after the split from non-African populations (71,78,80,81), hinting at survival of archaic human populations until relatively recently in time. This contrasts with observations from the fossil record, where most of the morphological diversity disappeared before 100 000 ya.

### Future Outlook

One of the questions that are still unresolved is how *Homo sapiens* arose. While it is clear that human origin, or emergence, is an evolutionary process over a relatively long time, we can narrow down the question of how humans originated by setting some time-limits. Starting from the present and going backward in time, the deepest divergence among humans—some 300 000 years ago—can be viewed as lower bound for the existence of fully developed (likely both behavioral and cognitive) modern humans (but not necessarily anatomically modern humans) simply because the descendant groups (that live today) of this first divergence certainly are modern humans. The human Neandertal/Denisovan divergence of some 650 000 years into the past can then be viewed as an upper bound of the development of unique features of *Homo sapiens.* In other words, by using these constraints, we can ask the question how humans developed unique characteristics from the time of the common ancestor of Neandertals/Denisovans till the starting time of human stratification observed today. Such unique characteristics may be governed by genetic changes, including frequency changes, and potentially epigenetic changes. There are various models of the origin of our species in Africa. Some of these include versions of (i) a single origin that expands and replaces all other hominin populations, (ii) an African multiregional model, where several African groups (likely geographically separated) are deeply stratified, possibly by isolation and (iii) range expansion from one group/area, but with some regional continuity and/or archaic introgression (83). The diverse morphology from fossil finds, material culture and genetics are hinting against a purely single origin of modern *Homo sapiens* in Africa (5,29,30), but possibly for emergence from a scattered diverse population with sporadic gene-flow (30,58). However, it is unclear how a model of a structured metapopulation can both uphold sufficient barriers to gene-flow to cause stratification while allowing for genetic variants important to modern human development to spread among populations simultaneously.

Disentangling the potentially complex early history of humans is a challenging task as later events, including various forms of gene-flow and migration, can distort genetic signals. More complex models, more diverse data and better statistical tools will allow us to look beyond simple bifurcating models and facilitate disentangling the intricate demography of African populations and inform on the models of the origin of our species as outlined in the previous paragraph. With efforts to gather more data, we still see a bias in data sampling; southern Africa in particular is currently overrepresented, both for modern and ancient genomic data. More genomic data from understudied regions and potential improvements in analyzing ancient DNA will refine our geographic and temporal understanding of African prehistory and allow us to decipher the early events.
